# Toxicity Evaluation of TiO_2_ Nanoparticles on the 3D Skin Model: A Systematic Review

**DOI:** 10.3389/fbioe.2020.00575

**Published:** 2020-06-10

**Authors:** Priscila Laviola Sanches, Luths Raquel de Oliveira Geaquinto, Rebecca Cruz, Desirée Cigaran Schuck, Márcio Lorencini, José Mauro Granjeiro, Ana Rosa Lopes Ribeiro

**Affiliations:** ^1^Postgraduate Program in Translational Biomedicine, University of Grande Rio, Duque de Caxias, Brazil; ^2^Directory of Metrology Applied to Life Sciences, National Institute of Metrology, Quality and Technology, Duque de Caxias, Brazil; ^3^Postgraduate Program in Biotechnology, National Institute of Metrology Quality and Technology, Duque de Caxias, Brazil; ^4^Fluminense Federal University, Rio de Janeiro, Brazil; ^5^Pesquisa e Desenvolvimento, Grupo Boticário, Curitiba, Brazil

**Keywords:** titanium dioxide, nanoparticles, 3D skin model, alternative method, toxicity

## Abstract

Titanium dioxide nanoparticles (TiO_2_ NPs) are regularly used in sunscreens because of their photoprotective capacity. The advantage of using TiO_2_ on the nanometer scale is due to its transparency and better UV blocking efficiency. Due to the greater surface area/volume ratio, NPs become more (bio)-reactive giving rise to concerns about their potential toxicity. To evaluate the irritation and corrosion of cosmetics, 3D skin models have been used as an alternative method to animal experimentation. However, it is not known if this model is appropriate to study skin irritation, corrosion and phototoxicity of nanomaterials such as TiO_2_ NPs. This systematic review (SR) proposed the following question: Can the toxicity of TiO_2_ nanoparticles be evaluated in a 3D skin model? This SR was conducted according to the Preliminary Report on Systematic Review and Meta-Analysis (PRISMA). The protocol was registered in CAMARADES and the ToxRTool evaluation was performed in order to increase the quality and transparency of this search. In this SR, 7 articles were selected, and it was concluded that the 3D skin model has shown to be promising to evaluate the toxicity of TiO_2_ NPs. However, most studies have used biological assays that have already been described as interfering with these NPs, demonstrating that misinterpretations can be obtained. This review will focus in the possible efforts that should be done in order to avoid interference of NPs with biological assays applied in 3D *in vitro* culture.

## Introduction

The development of the nanotechnology area, concerning to the nanomaterials production, is in exponential growth (Wang and Tooley, 2011). According to the International Organization for Standardization (ISO), nanomaterial is defined as natural, incidental or manufactured material containing particles (unbound, aggregated, agglomerated state), where 50% or more of the particles, have one or more external dimensions in the size range between 1 and 100 nm (Potočnik, 2011; International Organization for Standardization, 2017).

Due to their nano dimensions, they can effectively have electrical, thermal, and mechanical features, desirable for several applications (Davis et al., 2010; Louro et al., 2019; Lüderwald et al., 2019). Consequently, the human exposure to nanoparticles (NPs) increased as a result of their use in industries such as: food, pharmaceutical, cosmetic, biomedical (medical devices: implants, prostheses, controlled drug delivery systems), aeronautics, textiles as well as environmental engineering (Kongsong et al., 2014; Sethi et al., 2014; Semenzin et al., 2015; Miyani and Hughes, 2016; Hanawa, 2019; Shetti et al., 2019).

Regarding cosmetics, it is already known that many products contain various types of nanometric materials such as: gold, zinc oxide, titanium dioxide, nanotubes, fullerenes, among others (Morganti, 2010). Some of the referred nanostructures were introduced in sunscreens, with the final goal of protecting skin from solar radiation, reducing the chances of melanoma and also early skin aging (Wolf et al., 2001; Rampaul et al., 2007). NPs are among the best photoprotective agents since they are able to block the ultraviolet radiation incidence (González et al., 2008). Currently, titanium dioxide nanoparticles (TiO_2_ NPs) are the nanostructures mostly used in commercially available sunscreens, due to their ability to reflect and spread ultraviolet A (UVA, 320–400 nm) and ultraviolet B (UVB, 290–320 nm) rays, protecting against sunburn and photoaging (Monteiro-Riviere et al., 2011; Martirosyan and Schneider, 2014). TiO_2_ was previously classified as an inert particle, unable to be absorbed by the skin (Nohynek et al., 2007). When these sunscreens were created, TiO_2_ was used in the micrometric scale, being visible in the skin as an opaque layer. With the advancement of nanotechnology and aiming to solve this undesirable visual effect, TiO_2_ NPs were introduced in the formulations. The EU's Scientific Committee on Consumer Safety (SCCS) approved nanometric titanium dioxide (in the three crystalline forms) to be considered safe for use in cosmetic products intended for application on healthy, intact or sunburnt skin. As UV filter it can be introduced in cosmetic formulations at a maximum concentration of 25%, except in applications that may lead to exposure of the end user's lungs by inhalation. The benefits of using TiO_2_ NPs is their high surface area, increased properties of scattering and reflection of ultraviolet rays and transparency in visible light (Wiesenthal et al., 2011). In contrast, this size reduction of TiO_2_, increases their chances of internalization by skin cells with possible biological consequences to consumers.

Some *in vitro* literature already described that TiO_2_ NPs induce toxicity, inflammation and genetic modifications that is enhanced upon UVA and UVB exposure (Jin et al., 2011; Shi et al., 2013; Tucci et al., 2013; Zhao et al., 2013; Wang et al., 2014). The possible mechanisms of toxicity include oxidative stress, where TiO_2_ NPs trigger the formation of reactive oxygen species (ROS) in different dermal cell lines (Tucci et al., 2013; Zhao et al., 2013; Wang et al., 2014; Foroozandeh and Aziz, 2015). ROS involvement in oxidative DNA damage, in human epidermal, HaCaT cells (Shukla et al., 2014) and human dermal fibroblasts (Saquib et al., 2012) was already reported. Resuming dermal toxicity is associated with ROS generation, oxidative stress, and collagen depletion that promote skin aging (Wu et al., 2009). Actually, the strategy in the sunscreen industry is to coat nano-TiO_2_ in order to minimize their potential toxicity (Dréno et al., 2019). Compounds such as: silica, alumina, cetylphosphate, manganese dioxide, triethoxycaprylylsilane, PEG among others, contribute to making sunscreens more passive, improving their ability of capture or inhibit the formation of free radicals' species as well as to restrain NPs penetration in the skin (Filipe et al., 2009; Osmond and McCall, 2010; Smijs and Pavel, 2011). These alterations in NPs surface characteristics give rise to the need of a new set of physicochemical characterization, *in vitro* and *in vivo* evaluation, since surface modification regulates both inter-particle and cell-NP interactions, mediating corona formation that is widely known to induce specific cellular responses such as cellular uptake, intracellular trafficking, accumulation and biodistribution (Oberdörster et al., 2005; Filipe et al., 2009; Osmond and McCall, 2010; Foroozandeh and Aziz, 2015; Ribeiro et al., 2017; Sanches et al., 2019). Results suggest that benefitting from a physical barrier in the form of a coating minimizes ROS formation and consequent dermal toxicity (Yu et al., 2020).

The *in vitro* and *in vivo* data regarding the potential of dermal absorption and/or penetration of TiO_2_ NPs from sunscreens exhibit controversial results. Although several articles describe the opposite (Filipe et al., 2009; Senzui et al., 2010; Crosera et al., 2015), the penetration of TiO_2_ NPs in healthy as well as in damaged or lesioned skin (such as in cases of scarring, sunburn and depilated skin) is demonstrated in the scientific community (Tan et al., 1996; Lekki et al., 2007; Gontier et al., 2008; Schneider M. et al., 2009; Lin et al., 2011; Monteiro-Riviere et al., 2011; Larese Filon et al., 2013; Gulson et al., 2015; Shakeel et al., 2016; Touloumes et al., 2020), with damaged or lesioned skin being more susceptible to TiO_2_ NPs penetration (Tan et al., 1996; Lekki et al., 2007; Gontier et al., 2008; Schneider M. et al., 2009; Lin et al., 2011; Monteiro-Riviere et al., 2011; Larese Filon et al., 2013; Gulson et al., 2015; Shakeel et al., 2016; Touloumes et al., 2020). Prolonged application of TiO_2_ NPs sunscreens in healthy human skin, reveal the detection of titanium levels in the epidermis and dermis of patients (Tan et al., 1996; Lin et al., 2011; Gulson et al., 2015; Næss et al., 2015; Shakeel et al., 2016). Recently, although the study has some limitations (few number of volunteers), Pelclova et al. using highly sensitive characterization techniques detected TiO_2_ NPs in plasma and urine after 6 to 48 h after sunscreen exposure, demonstrating that TiO_2_ NPs can pass the healthy protective layers of human skin and enter in blood circulation, even with lower exposure times of sunscreen application (Pelclova et al., 2019). The penetration of NPs is not exclusive to TiO_2_ NPs, with Brian Gulson et al. reporting also the detection of zinc oxide used in sunscreens in human blood and urine (Gulson et al., 2010). From our point of view, and also already stated by OECD report that evaluate the *in vitro* methods for human hazard assessment of nanomaterials (Organisation for Economic Co-operation Development, 2018), there exist many critical points in the available literature that contribute to all the controversy regarding TiO_2_ NPs penetration in human skin. It is important to refer that several factors such as: the model employed (animal, human with variations in gender), size, chemical composition and coating of NPs, site of application, particle solubility, dose and number of applications, period of the study, the flexion motion of skin, UV exposure among others, influence the dermal penetration of nanoparticles (Gulson et al., 2015; Shakeel et al., 2016). To the best of our knowledge, most of the studies are performed with a strong variety of conditions and methodologies, where no standardized protocols and reference nanomaterials are used. More important is that the characterization techniques used to evaluate NPs skin penetration were sometimes entering in the detection limit of the equipment, contribute to all this controversy.

**Figure 1:**
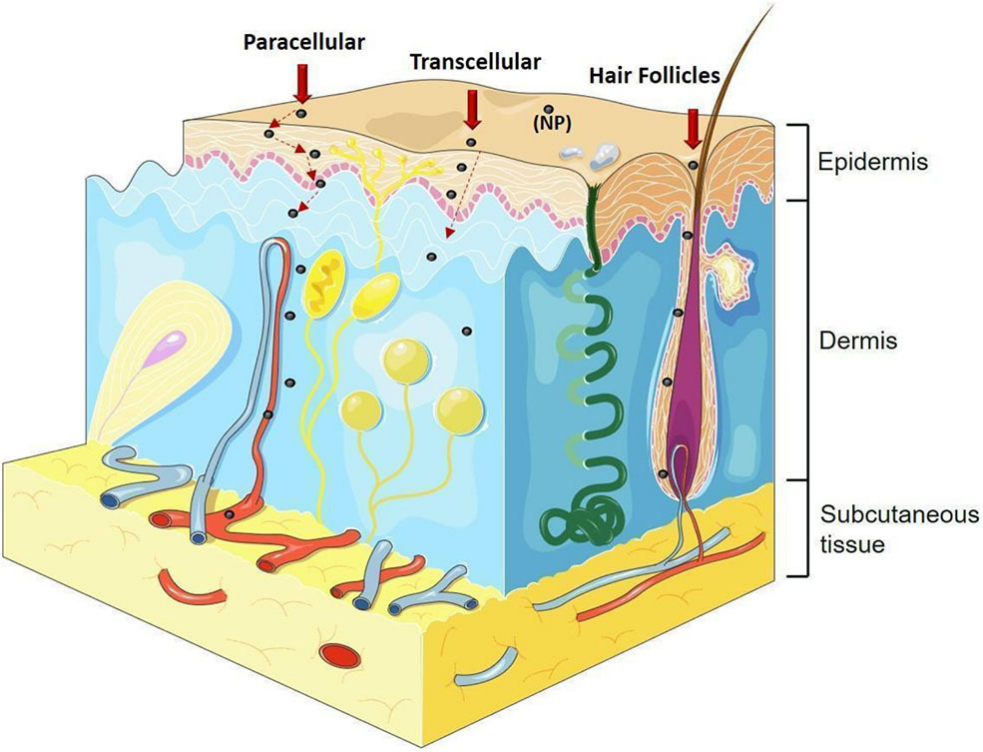
Different pathways of nanoparticle penetration. Paracellular transport (between cells), transcellular transport (inside the cells), transport by hair follicles, sweat glands, skin folds or a combination of all. Image adapted from: https://smart.servier.com.

The mechanism of penetration of sunscreen nanoparticles was not clarified, however, it is suggested that TiO_2_ NPs can be absorbed follow different pathways that include the transcellular and paracellular transports, as well as hair follicles (transappendageal), sweat glands, skin folds or a combination of all, as shown in Figure 1 (Filipe et al., 2009; Wu et al., 2009). The fact that upon skin penetration, NPs can reach the bloodstream, and then undergo translocation to various distant tissues and organs, suggest that prolonged time exposure to NPs, may pose a health risk to consumers (Lademann et al., 1999; Baroli et al., 2007; Lekki et al., 2007; Saquib et al., 2012; Shakeel et al., 2016).

**Figure 2:**
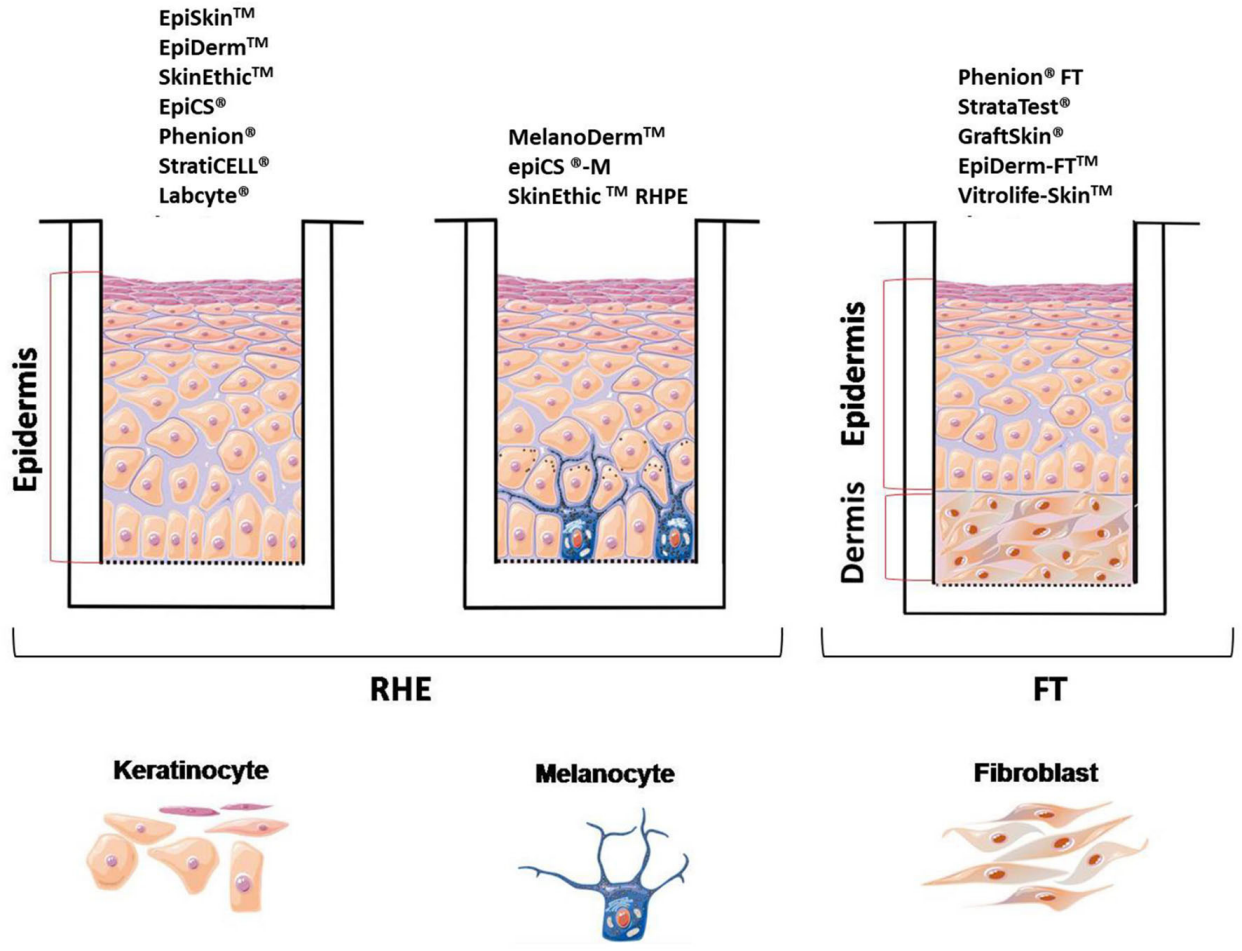
Scheme of the main 3D skin models used. Image adapted from https://smart.servier.com.

As previously described, there are already many *in vitro* (using 2D models) and *in vivo* (using animals) studies on the cytotoxicity and genotoxicity effect of TiO_2_ NPs. However, cosmetic industries are using alternative methods, such as 3D skin reconstructed models, due to ethical, scientific and economic considerations. The EU cosmetic legislation is working toward the abolition of animal testing for cosmetics and their ingredients (Evans et al., 2016; Salamanna et al., 2016; Caddeo et al., 2017; Alépée et al., 2018; Owen et al., 2018). In fact, 3D engineered models mimicking human tissues are under development to overcome the limitations of 2D *in vitro* models regarding their limited predictivity (Vernetti et al., 2017). Currently, there are commercially available 3D skin models as well as in-house constructs with several levels of biological complexity. Figure 2 exhibits some of the commercially available models.

The simplest model consists of an epidermis where only keratinocytes are used, it is known as reconstructed human epidermis (RHE) and it is commercially available as EpiDerm™ (MatTek Corp), EpiSkin™ and SkinEthic™ (a subsidiary of L'Oreal). They make use of reconstructed human epidermis, which closely mimics the histological, morphological, biochemical, and physiological properties of the epidermal layer of human skin (Kim et al., 2016). The advantage of RHE models is that they contain all the epidermal layers of skin, with the main disadvantage of, it is not always possible to distinguish the basale, spinosum and granulosum stratum, important issue for penetration studies. Also, a low intra-batch variation and sometimes a high inter-batch variation is observed (Mathes et al., 2013; Almeida et al., 2017). In April 2007, EpiSkin™ and Epiderm™ models were approved by ECVAM (European Center for the Validation of Alternative Methods) to replace *in vivo* rabbit skin irritation test. They are also used for other regulatory purposes, such as *in vitro* skin irritation (OECD TG 439) and skin corrosion (OECD TG 431) tests of cosmetic ingredients. An update in 2019 was made to include two additional models namely SkinEthic™ and epiCS^®^ (OECD, 2014, 2019). Besides that, these models are widely used for phototoxicity, genotoxicity, sensitization, metabolization tests as well as to test the administration of transdermal drugs (Mathes et al., 2013; Almeida et al., 2017). EpiSkin, for example, is an *in vitro* reconstructed human epidermis from normal human keratinocytes cultured on a collagen matrix at the air-liquid interface that is validated for skin corrosion/irritation studies (Alépée et al., 2018; Liu et al., 2018). Cell viability is the main endpoint, however complementary assays can be performed, such as gene array, histological, morphological analysis and cytokine release (Sarmento et al., 2012; Almeida et al., 2017). The Full-Thickness Skin Model (FT), commercially known as EpiDerm-FT™ is a more elaborated model that consists of an epidermis and dermis (keratinocytes and fibroblasts) and has been widely used in drug or efficacy treatments (Mathes et al., 2013). This model has the advantage of providing wall-to-wall tissue, as well as having a basal membrane similar to *in vivo* when compared to RHE models (simplest model). The permeability of RHE models is inferior to human and pig skin, however they are accepted to test in *vitro* permeation and penetration studies when drugs are applied as aqueous solutions (Asbill et al., 2000; Schäfer-Korting et al., 2008; Neupane et al., 2020). Resuming for *in vitro* skin corrosion tests following OECD TG 431, the commercially available models are: EpiSkin^TM^ Standard Model (SM), EpiDerm^TM^ SCT, SkinEthic^TM^ RHE, epiCS^®^, and LabCyte EPI-MODEL24 SCT (OECD, 2014). Regarding *in vitro* skin irritation following TG 439 the used models are: EpiSkin^TM^ (SM), EpiDerm^TM^ (SIT), SkinEthic^TM^ (RHE), LabCyte EPI-MODEL24 SIT, epiCS^®^ and Skin+ ^®^ (OHAT, 2015). In order to test sun care products companies are also developing 3D skin models incorporating melanocytes (e.g., MelanoDerm^TM^, MatTek corp.; epiCS^®^-M, ATERA SAS & CellSystems Gmbh; and SkinEthic^TM^ RHPE subsidiary of L'Oréal). Although 3D skin models have been used by the cosmetic industry for corrosion and skin irritation testing of chemical formulations, to the date it is not known whether this models are appropriate for studying the cytotoxicity, skin corrosion, irritation, and phototoxicity of formulations containing TiO_2_ NPs. For this reason, this systematic review aims to answer the following proposed question: Can the toxicity of TiO_2_ nanoparticles be assessed in the 3D skin model?

## Materials and Methods

This systematic review (SR) was conducted in accordance with the *Cochrane Handbook for Systematic Reviews of Interventions* (Higgins and Green, 2011), the Preferred Reporting Items for Systematic Review and Meta-Analyses (PRISMA) guidelines (Moher et al., 2009) and the Office of Health Assessment and Translation (OHAT) handbook (OHAT, 2015), which was developed by National Toxicity Program. The protocol of this SR was registered in CAMARADES at http://www.dcn.ed.ac.uk/camarades/research.html#protocols. Also, a reliability assessment of *in vitro* toxicity studies named Toxicological data Reliability Assessment Tool (ToxRTool), was followed to increase the quality and transparency of this search.

### Focused Question (Based on PICO Strategy) (Schardt et al., 2007)

- Population 3D skin model
- Interventions or exposure of TiO_2_ NPs in the 3D model
- Comparison 3D model without TiO_2_ NPs exposure
- Outcome Effects generated by TiO_2_ NPs in the 3D skin model: cytotoxicity, phototoxicity, irritation, corrosion.
- Study design *in vitro* studies.

### Search Strategy

An electronic search was carried out in the MEDLINE/PubMed, Science Direct, Web of Science, Scopus and SciELO library databases up to February 2019. Only studies in English, Portuguese, Spanish, or French were selected, without date restriction. In addition, searches in the references of the included studies (i.e., cross-referencing) were conducted. Furthermore, unpublished studies (i.e., gray literature) were analyzed in the Gray Literature Report and OpenGrey databases. A specific search strategy was used for each database, according to its characteristics (Table 1).

**Table 1:** Search strategy.

**Databases**	**Keywords**
PubMed	(“3d skin model”[tiab] OR “reconstructed human skin model”[tiab] OR “human skin model”[tiab] OR “epidermis model”[tiab] OR “episkin”[tiab] OR “epidermis”[tiab] OR “skin equivalent model”[tiab] OR “reconstructed human epidermis”[tiab]) AND (“titanium dioxide”[tiab] OR “tio2”[tiab] OR “titanium”[tiab] OR “titanium”[mesh]) AND (“nanoparticles”[tiab] OR “NP”[tiab] OR “nanomaterials”[tiab] OR “nanoparticles”[Mesh] OR “metal nanoparticles”[mesh]) AND (“skin corrosion”[tiab] OR skin “irritation”[tiab] OR “toxicity”[tiab] OR “cytotoxicity”[tiab] OR “phototoxicity”[tiab] OR “irritation”[tiab] OR “corrosion”[tiab] OR “Skin Irritancy Tests”[Mesh])
Science Direct	(“skin model” OR epidermis OR episkin OR “skin equivalent model” OR “reconstructed human epidermis”) AND (titanium OR tiO2) AND (nano*) AND (corrosion OR irritation OR toxicity OR cytotoxicity OR phototoxicity)
Web of science	(“skin model” OR “epidermis model” OR “episkin” OR “skin equivalent model” OR “reconstructed human epidermis”) AND (“tio2” OR “titanium”) AND (“nanoparticles” OR “NP” OR “nanomaterials”) AND (“skin corrosion” OR “skin irritation” OR “toxicity” OR “cytotoxicity” OR “phototoxicity” OR “irritation” OR “corrosion”)
Scopus	(“skin model” OR “epidermis model” OR “episkin” OR “epidermis” OR “skin equivalent model” OR “reconstructed human epidermis”) AND (“tio2” OR “titanium”) AND (“nano*”) AND (“toxicity” OR “citotoxicity” OR “phototoxicity” OR “irritation” OR “corrosion”)
Scielo	(“skin model” OR “epidermis model” OR “episkin” OR “epidermis” OR “skin equivalent model” OR “reconstructed human epidermis”) AND (“tio2” OR “titanium”) AND (“nano*”) AND (“toxicity” OR “citotoxicity” OR “phototoxicity” OR “irritation” OR “corrosion”)
Gray Literature	(skin OR epidermis) AND (titanium OR tio2) AND (skin irritancy test OR *toxicity)

### Eligibility Criteria (OHAT)

The inclusion and exclusion criteria are stated in Table 2.

**Table 2:** Inclusion and exclusion criteria.

**PECO**	**Inclusion**	**Exclusion**
Population	*In vitro* study in 3D skin model	Clinical trials *In vivo* studies *In vitro* studies of monolayer culture
Exposure	Exposure of TiO_2_ NPs TiO_2_ must have nanometric size TiO_2_ suspensions in any crystalline phase or mixture	Average size in micrometric scale
Comparison	3D model without NPs exposure	
Outcome	Effects generated by NPs in the 3D skin model: cytotoxicity, phototoxicity, irritation, and corrosion	Studies focusing on the characterization and synthesis of NPs
Publication type	Reports must contain original data	Articles with no original data (e.g., editorials, reviews, letters) Languages other than Portuguese, Spanish and English Studies published in abstract form only Book chapters

### Study Selection, Screening Process, and Data Extraction

Titles and abstracts of the retrieved articles were screened by two authors/reviewers (PS and LG) and then publications that fulfilled the inclusion criteria were identified. Disagreements between the reviewing authors were resolved through careful discussion, and any remaining disagreements were resolved by a third reviewer (JMG). After that, full text of eligible articles was obtained. Finally, based on the inclusion criteria two authors/reviewers (PS and LG) independently screened and selected the relevant full-text articles. Furthermore, disagreements between the reviewing authors were resolved through the same process used in the first selection phase.

When available, the following data were extracted from the publications by the reviewers (PS and RC): year, DOI, type of skin model, test substance, the crystalline structure of TiO_2_ NPs, primary particle size, size of the TiO_2_ after dispersion, exposure time, the expected outcome of the positive and negative controls, results in the evaluation of corrosion and/or irritation and phototoxicity and main conclusion.

### Assessment of Reliability

The reliability assessment was done by two reviewers (PS and LG), using the ToxRTool developed by the European Center for the Validation of Alternative Methods (ECVAM) (Schneider K. et al., 2009). The *in vitro* part of this tool consists of a list of 18 criteria. Each criterion can be graded as “1” (i.e., “criterion met”) or as “0” (i.e., “criterion not met” or not reported). Those 18 criteria are grouped in five major groups: *I*-Test substance identification, II-Test system characterization, III-Study design description, IV-Study results documentation and V-Plausibility of study design and results. A final score was then recorded for each major group of each article, and an overall score was recorded for each study.

In this tool, there are some criteria considered indispensable for a study to be reliable, and they are highlighted in red (presented in Supplementary Information). Independently of the overall score, only if these criteria are assigned as “1” the tool will rate the study as a reliable category (1 or 2). Those categories are: 1 (reliable without restrictions), 2 (reliable with restrictions), 3 (not reliable) and 4 (not assignable).

Finally, ToxRTool classified under “A” box the category in which the article was assigned based on the sum of points (overall score), regardless the red criteria. And, under “B” the category is derived considering the red criteria.

## Results

### Search

**Figure 3:**
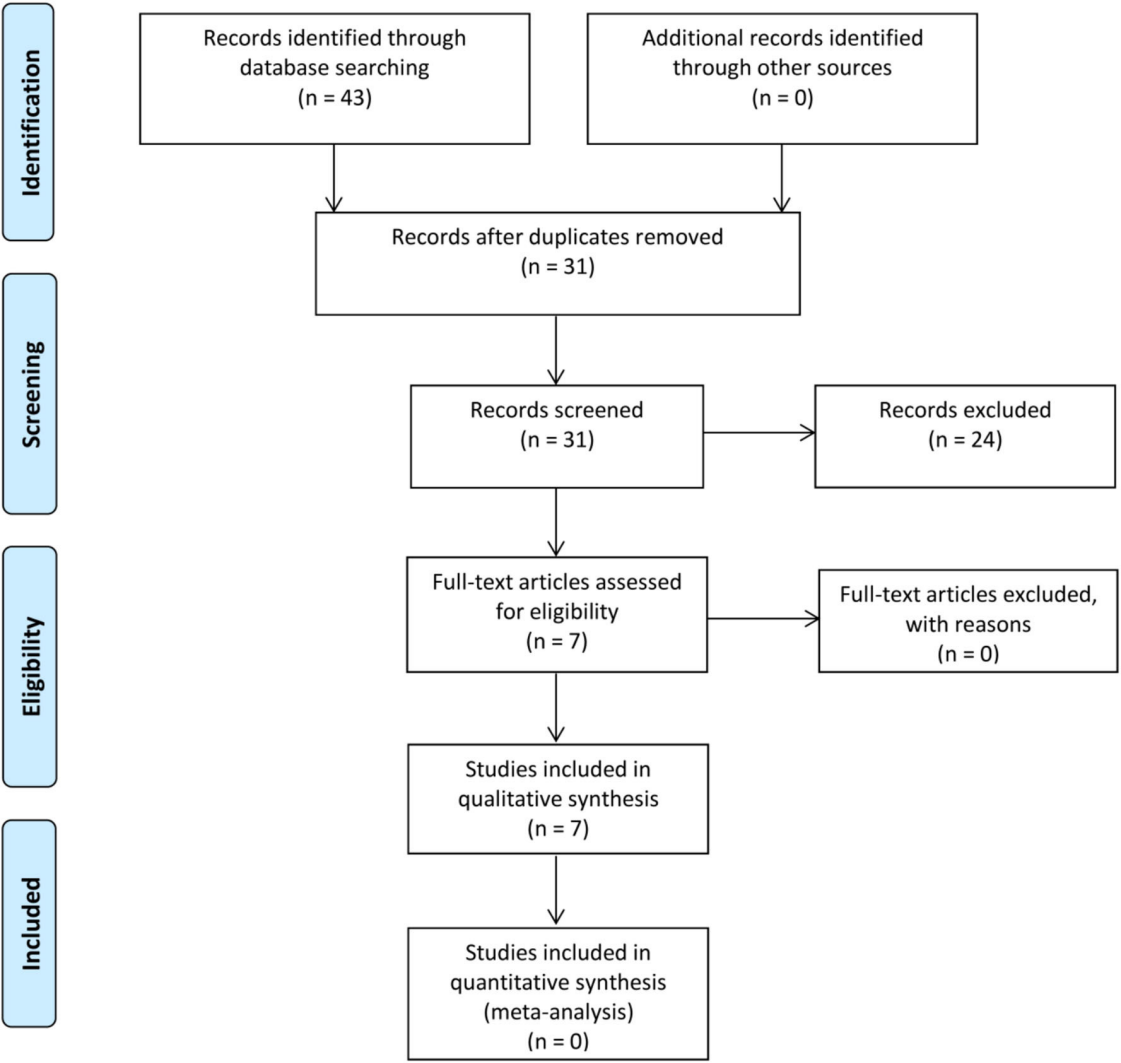
Flow diagram of literature selection process, from Moher et al. (2009).

The initial search identified 43 articles, comprising 11 titles from MEDLINE/PubMed, 13 from Science Direct, 12 from Scopus, and 7 from Web of Science. The search in the gray literature or cross-referencing did not yield any studies. After duplicates removal, 31 studies had their titles and abstracts screened, and 24 studies were excluded because they did not meet the eligibility criteria. Then, the full-text screening did not exclude any studies, resulting in 7 included articles (Park et al., 2011; Choi et al., 2014; Kato et al., 2014; Horie et al., 2016; Kim et al., 2016; Miyani and Hughes, 2016; Tang et al., 2018) in this systematic review (Figure 3 Prisma Flow diagram).

### Assessment of Reliability

As described previously, the reliability assessment was done using the ToxRTool tool. It can be observed that a few criteria were not met by the authors. The number of replicates and the statistical methods for data analysis given is some of the criteria that have not been described by some authors. In Table 3 it is possible to observe the general punctuation and categories of each selected article. Thus, all articles were classified in category A with “1,” which corresponded to that the articles that were considered as reliable without restrictions. Articles were also classified in category B with “1” when all criteria considered indispensable for the study was reliable and attended.

**Table 3:** General punctuation and categories of the selected articles.

**References**	**Overall score**	**Category A**	**Category B**
Park et al. (2011)	16	1	1
Choi et al. (2014)	16	1	1
Kato et al. (2014)	17	1	1
Horie et al. (2016)	16	1	1
Miyani and Hughes (2016)	16	1	1
Kim et al. (2016)	17	1	1
Tang et al. (2018)	18	1	1

### Study Characteristics

From the seven articles found and analyzed, four studies used EpiDerm™ (MatTek Corporation) (Park et al., 2011; Horie et al., 2016; Kim et al., 2016; Miyani and Hughes, 2016), one used the EpiKutis™ (Biocell Biotechnology, China) (Tang et al., 2018), one used KeraSkin (Modern Cell & Tissue Technology, Seoul, Korea) (Choi et al., 2014) and the last one was developed in their own laboratory (Kato et al., 2014). According to the inclusion criteria, all seven articles used TiO_2_ NPs as test substance. As it is well known, TiO_2_ NPs exhibits three crystalline forms: rutile, anatase, and brookite. However, some studies also use mixtures of anatase and rutile structures. From the selected articles, two articles used the rutile crystalline structures (Choi et al., 2014; Kato et al., 2014), two articles used the mixture (anatase and rutile) (Park et al., 2011; Kim et al., 2016), one article used two crystalline structures, anatase and rutile (Horie et al., 2016), and two articles used anatase, rutile, and mixture (Miyani and Hughes, 2016; Tang et al., 2018). Some authors have also used other test substances such as: NPs of zinc oxide, zinc oxide/titanium dioxide (Choi et al., 2014), silver (Kim et al., 2016; Miyani and Hughes, 2016), cerium dioxide (Miyani and Hughes, 2016), iron oxide (Kim et al., 2016), aluminum oxide (Kim et al., 2016), polystyrene (Park et al., 2011) and polyvinylpyrrolidone-entrapped fullerene-C_60_ (Kato et al., 2014).

The primary size of the TiO_2_ NPs ranged from 6 to 108 nm. Regarding the size of agglomerates after dispersion, as it can be seen in Table 4, only three authors precisely described NP_S_ size (Choi et al., 2014; Horie et al., 2016; Tang et al., 2018), two authors did not describe the size (Kato et al., 2014; Miyani and Hughes, 2016), one author demonstrated the agglomerate size by transmission Electron Microscopy image (which is apparently around 200 nm (Park et al., 2011) and one author states that the size of NPs was several hundred nanometers (Kim et al., 2016).

**Table 4:** Extraction of data from selected articles.

**Authors**	**Park et al. (2011)**	**Choi et al. (2014)**	**Kato et al. (2014)**	**Horie et al. (2016)**	**Miyani and Hughes (2016)**	**Kim et al. (2016)**	**Tang et al. (2018)**
Year	2011	2014	2014	2016	2016	2016	2018
DOI	10.1016/ j.tiv.2011.05.022	10.5620/ eht.2014.29.e2014004	10.1166/ jnn.2014.8719	10.1080/15376516. 2016.1175530	10.1080/15569527. 2016.1211671	10.5487/ TR.2016.32.4.311	10.1016/j.tox. 2018.05.010
Type of skin model	EpiDerm™	KeraSkin™	Developed in own laboratory	EpiDerm™	EpiDerm™	EpiDerm™	EpiKuitis™
Test substance	Polystyrene and Titanium dioxide nanoparticles	Zinc oxide nanoparticles, Titanium dioxide nanoparticles and Mixture (Zinc oxide/Titanium dioxide nanoparticles)	Polyvinylpyrrolidone-entrapped fullerene-C60 and Titanium dioxide nanoparticles	Titanium dioxide nanoparticles	Silver nanoparticles, Titanium dioxide nanoparticles and Cerium dioxide nanoparticles	Iron nanoparticles, Aluminum oxide nanoparticles, Titanium oxide nanoparticles and Silver nanoparticles	Titanium dioxide nanoparticles
Crystalline structure of TiO_2_	Mixture	Rutile	Rutile	Anatase and Rutile	Anatase, Rutile and Mixture	Mixture	Anatase, Rutile and Mixture
Primary particle size	25 nm	21 nm	10 nm	Anatase: 6 nm and 7 nm; Rutile: 15 nm	Anatase: 25 nm and 142 nm; Rutile: 214 nm; Mixture: 22 nm, 31 nm and 59 nm	21 nm	Anatase: 23 nm and 108 nm, Rutile: 21 nm and Mixture: 31 nm, 52 nm and 55 nm
Size of the TiO_2_ after dispersion (aggregate)	± 200 nm	39.4 ± 28.6 nm	Not described	Anatase: 252.1 nm and 323.2 nm; Rutile: 276.4 nm and 318.4 nm	Not described	Several hundred nm in size	Anatase: 912 nm and 410 nm, Rutile: 121nm and Mixture: 2312 nm, 246 nm and 249 nm
Analysis method	Skin irritation test; skin phototoxicity test	Skin Corrosion Test; Skin Irritation Test; Cytokine Assay and Histopathology	Intracellular ROS-Generation and Lipid Hydroperoxides	Lactate dehydrogenase assay, IL-8 enzyme-linked immunosorbent assay and relative HO-1 gene expression; Skin Irritation Test	Skin irritation test	Skin Corrosion Test; Skin Irritation Test; IL-1α assay and histopathology	Phototoxicity test
Substance concentration of TiO_2_	100 μg/mL	25% in deionized water	15 μg/mL	100 μg/mL	1 mg/ml	The epidermis surface was moistened with deionized water and 25 mg of the test substance was added	100 μg/mL
Exposure time	1 h	3 min and 1 h	3 h	4 h	1 h	3 min and 1 h	2 h
The expected outcome of the positive and negative controls was observed?	Yes	Yes	Yes	Yes	Yes	Yes	Yes
Results evaluation of corrosion and/or irritation and phototoxicity	Cytotoxicities and phototoxicity were assessed as a percentage of the negative control. However, the negative control exhibits the viability > 50%.	Corrosion: The article uses OECD TG431 as a reference, which says that the test material is considered to be corrosive to the skin if the viability is <50% after 3 min exposure. However, although the viability after 3 min exposure is ≥ 50%, it is corrosive if the viability is <15% after 60 min exposure. Therefore, the material is non-corrosive if the viability is ≥ 50% after 3 min exposure and ≥ 15% after 60 min exposure. Irritation: The article uses OECD TG439 as a reference, which says that test material is considered to be irritant to skin if the tissue viability after exposure/post-incubation is ≤ 50%, and is non-irritant if the viability is > 50%.	Evaluation of the induction of ROS-generation around the outside of nuclei and peroxidation of cell membrane in the epidermis by electron microscopy.	Irritation: The article uses OECD TG439 as a reference, which says that test material is considered to be irritant to skin if the tissue viability after exposure/post-incubation is ≤ 50%, and is non-irritant if the viability is > 50%.	Regarding the irritation test, the test material is considered to be irritant to skin if the tissue viability after exposure/post-incubation is less than or equal to 50%. It would be non-irritant if the viability is more than 50%.	Corrosion: The article uses OECD TG431 as a reference, which says that the test material is considered to be corrosive to the skin if the viability is <50% after 3 min exposure. However, although the viability after 3 min exposure is ≥ 50%, it is corrosive if the viability is <15% after 60 min exposure. Therefore, the material is non-corrosive if the viability is ≥ 50% after 3 min exposure and ≥ 15% after 60 min exposure. Irritation: The article uses OECD TG439 as a reference, which says that test material is considered to be irritant to skin if the tissue viability after exposure/post-incubation is ≤ 50%, and is non-irritant if the viability is > 50%.	The test substance was considered phototoxic if the UVA exposed tissues revealed a decrease in viability exceeding 25% when compared with the dark control.
Main conclusion	Polystyrene and Titanium dioxide nanoparticles did not exhibit skin irritation and phototoxicity	For all the test materials used in this study were found to be non-corrosive and non-irritant; The results were confirmed by IL-1α and histopathological analysis	Was observed that UV irradiation of 8 J/cm2 in the presence of 15 ppm TiO_2_ induced ROS-generation around the outside of nuclei and lipid peroxidation of cell membrane in the epidermis. However, the treatment with C60/PVP or C60/Sqn suppressed intracellular ROS and lipid peroxidation, and thereafter TiO_2_-catalyzed phototoxic potency was repressed in a dose-dependent manner of C60.	The UVA irradiation did not affect LDH leakage or IL-8 secretion in the culture medium, as no increase in HO-1 expression was observed, regardless of the type of TiO_2_ nanoparticle	The silver, cerium and titanium nanoparticles tested can be classified as non-irritants	For all the test materials used in this study were found to be non-corrosive and non-irritant; The results were confirmed by IL-1α and histopathological analysis	For all the test materials used in this study, there was no phototoxicity at test concentration in the studied skin model

The skin irritation test was performed in five articles. Two authors have done corrosion tests, and the other two performed phototoxicity tests. In addition to these tests, in some article's histopathology, cytokine assay, lactate dehydrogenase assay, IL-8 enzyme-linked immunosorbent assay, IL-1α assay, relative HO-1 gene expression, intracellular ROS-generation, and lipid hydroperoxides were performed.

Three authors used the concentration of 100 μg/mL, however, each author used different exposure times: 1 h (Park et al., 2011), 2 h (Tang et al., 2018) and 4 h (Horie et al., 2016). In addition, one author used the concentration of 15 μg/mL for 3 h of exposure (Kato et al., 2014), another used the concentration of 1 mg/mL for 1 h of exposure (Miyani and Hughes, 2016). Furthermore, two authors used the same exposure period of 3 min and 1 h. In one study the epidermis was moistened with deionized water and 25 mg of the test substance (Kim et al., 2016) and in another study, 25% of the test substance in deionized water was used (Choi et al., 2014).

### Toxicity Results

#### Skin Irritation

All articles that assessed skin irritation used 3- (4,5-dimethylthiazol-2-yl)−2,5-diphenyltetrazolium bromide (MTT) assay (Park et al., 2011; Choi et al., 2014; Kim et al., 2016; Miyani and Hughes, 2016). In all cases, TiO_2_ NPs was shown to be non-irritating on the 3D model, where no reduction in cell viability was observed. In all studies, the results were compared with the positive and negative controls, where Sodium Dodecyl Sulfate (SDS) was used as a positive control, Dulbecco's Phosphate Buffered Saline (DPBS) and Phosphate Buffered Saline (PBS) were used in some studies as a negative control.

#### Skin Corrosion

Two authors used TiO_2_ NPs to evaluate skin corrosion (Choi et al., 2014; Kim et al., 2016). Choi et al. and Kim et al., used potassium hydroxide (8N KOH) as a positive control (Choi et al., 2014; Kim et al., 2016). Choi et al. observed that after 3 min of exposure with 8N KOH the viability was reduced to 1%, while the TiO_2_ NPs treated sample viability was 94% (± 3.0). After 60 min of TiO_2_ NPs exposure, it was observed that the viability reduced in comparison to the time of 3 min, however, the viability was higher than 50% (Choi et al., 2014). Kim et al. presented similar results. They demonstrated that after 3 min of exposure, treatment with 25 mg of TiO_2_ NPs led to the viability of 96.3% (± 2.4) while 8N KOH reduced viability to 9.8% (± 1.6). After an exposure time of 60 min, the viability of the treated sample with TiO_2_ NPs decreased to 85.3% (±3.9) (Kim et al., 2016). Therefore, the two studies concluded that TiO_2_ NPs are non-corrosive, considering that viability was >50 and 15% after 3 and 30 min of exposure, respectively.

#### Phototoxicity

The assessment of TiO_2_ NPs phototoxicity in the 3D skin model was performed in two articles. It was applied variable exposition times, non-toxic doses of UVA of 6 J/cm^2^ (Park et al., 2011) and 40 J/cm^2^ (Tang et al., 2018). In both cases, phototoxicity was evaluated by the reduction of mitochondrial conversion of MTT to formazan. The authors concluded that TiO_2_ NPs did not exhibit phototoxicity in the 3D skin model in the presence of UV radiation.

## Discussion

With the advancement of nanotechnology, many products with nanoscale materials have been introduced in several areas such as cosmetics, food, drugs and electronics (Louro et al., 2019; Lüderwald et al., 2019). Therefore, exposure to nanomaterials is in exponential growth, and can occur both during the synthesis as well as in the use of the final product. Due to the greater surface area/volume ratio, NPs become more (bio) reactive compared to normal bulk materials, giving rise to concerns about their potential toxicity to humans (Sharifi et al., 2012; Shi et al., 2013).

TiO_2_ NPs are widely used in the cosmetic industries, especially in sunscreens as an alternative to available chemical UV absorbers (p-aminobenzoic acid and benzophenones) that are known to cause some allergic reactions and/or endocrine disruption. Titanium dioxide has three different crystalline structures (anatase, rutile and brookite), however, rutile is the most used in cosmetic, due to its high refraction index, protecting skin from the harmful effects of ultraviolet rays (Martirosyan and Schneider, 2014). The International Agency of Cancer Research (IARC) classified titanium dioxide as a possible human carcinogen (group 2B), however, there is no distinction regarding titanium size (macro, micron and/or nanoscale). The heterogeneity of TiO_2_ nanoparticles (particle size distribution; agglomeration and aggregation; morphology, crystal structure; purity) for sunscreen applications is high, becoming fiercely debated the hazard of TiO_2_ NPs (Jacobs et al., 2010).

A long time ago that cosmetic industries were testing their products using corrosion and skin irritation tests in rabbits (in accordance with the Organization for Economic Co-operation and Development (OECD) (OECD, 2015). However, due to ethical (3 R principle–replacement, refinement and reduction of animal trials), scientific and economic restrictions, skin bioengineering 3D tissues (Evans et al., 2016; Salamanna et al., 2016; Caddeo et al., 2017; Alépée et al., 2018; Liu et al., 2018; Owen et al., 2018) are actually used for testing new pharmaceuticals. The main advantages of the bioengineering 3D tissues are their physiological relevance since they recapitulate tissue's microenvironment, increased reproducibility comparing to *ex-vivo* models, superior predictive potential due to the possible use of human cells, along with decreased in cost and ethical concerns (Sarmento et al., 2012; Gholobova et al., 2018; Madl et al., 2018; Qiao and Tang, 2018). High quality skin constructs (that mimic the morphology, lipid composition and differentiation of native human skin) are commercially available. Current available 3D skin constructs still do not contain all essential cell types (dendritic cells as well as macrophages), neither integrate blood vessels, neither the dynamic crosstalk between epithelium and connective tissue, that are essential to regulate epidermal morphogenesis and homeostasis. This reinforces the need of more complex 3D constructs that can address complex toxicological endpoints. Nowadays the main strategy is to use biofabrication technique's such as electrospinning and bioprinting to develop relevant skin biological models constituted by specialized cell types (melanocytes, adipocytes, Langerhans, immune, stem cells, among others) with a perfused vasculature allowing a physiological oxygen and nutrient delivery (Mathes et al., 2013). The main goal is to achieve a physiological relevant 3D skin construct that can be used for toxicology assessment of new cosmetic formulations but also anti-cancer drugs development for example, reducing the animal studies.

A few articles study the hazard effect of TiO_2_ NPs in a three-dimensional skin model. Mostly, available literature uses quite diverse *in vitro* (primary cells and cell lines derived from different organs/tissues) and *in vivo* models to evaluate the hazard effect of TiO_2_ NPs. Results suggest that TiO_2_ NPs induce the release of ROS leading to loss of vital cellular functions, ultimately leading to cell death and accumulate in liver and spleen preferentially (Jin et al., 2011; Shi et al., 2013; Tucci et al., 2013; Zhao et al., 2013; Wang et al., 2014; Carriere et al., 2016; Proquin et al., 2016).

This systematic review demonstrates that the 3D skin models used, mimic the histology, morphological, physiological and biochemical properties of human epidermis. The reliability of these studies was evaluated by ToxRTool, and all 18 criteria evaluated by this tool are presented in Tables S2–S8 (Supplementary Information). This table describes a simulation of a work that meets 100% of the criteria as well as the evaluation performed in the seven selected articles (see Table S1).

**Figure 4:**
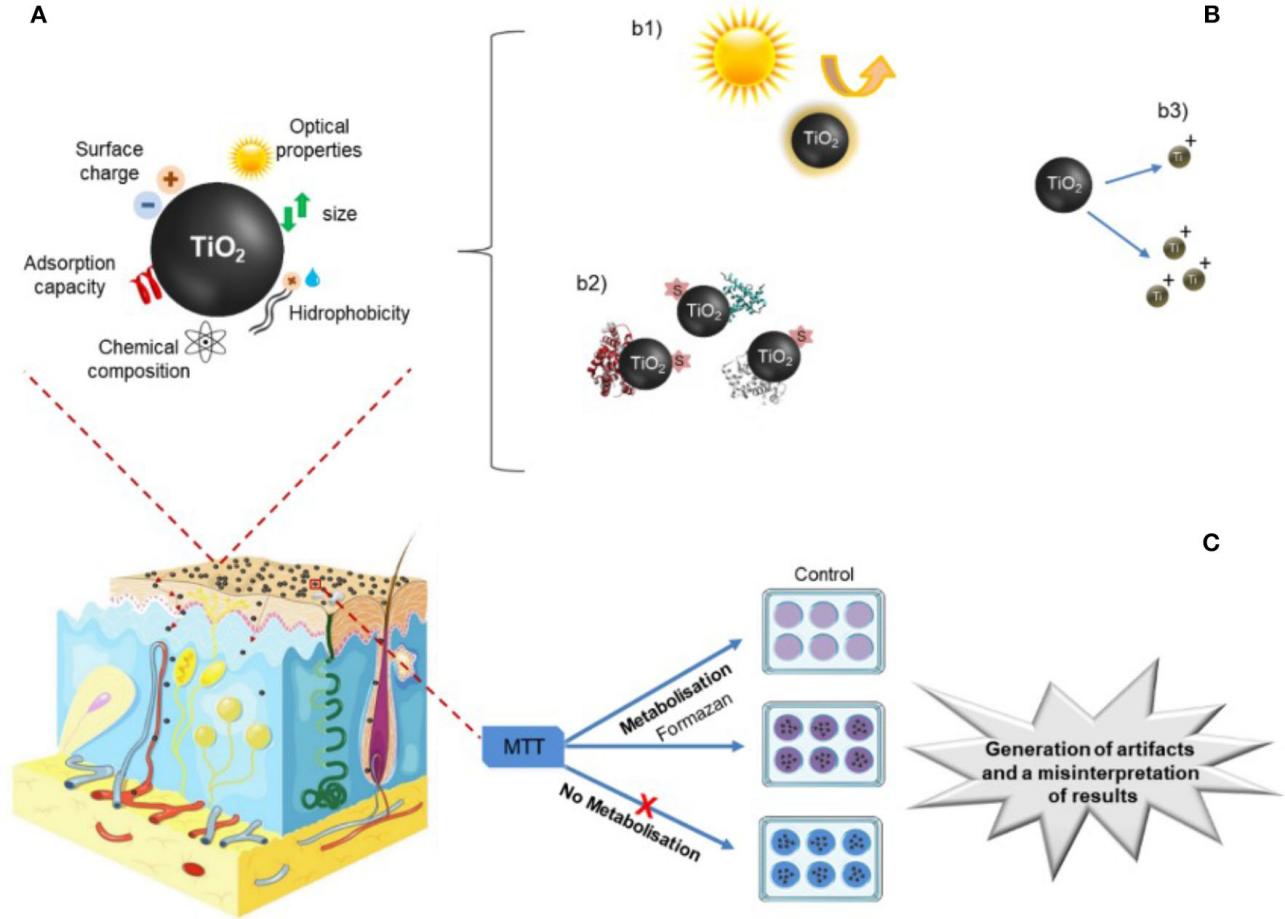
Toxicological assays. **(A)** Physico-chemical characteristics of NPs TiO_2_ NPs that possible interfere with the biological assays; that include **(B)** optical capabilities of TiO_2_ NPs such as intrinsic absorbance and/or fluorescence (b1); adsorption of proteins, salts and dyes (s) to NPs (b2); and dissolution of NPs with the consequent release of metal ions to supernatant (b3). **(C)** Conventional MTT test analyzing TiO_2_ NPs demonstrating that NPs can adsorb to MTT dye that avoid metabolization of reagents. Image adapted from: https://smart.servier.com.

As it can be observed in Table 3, all articles were well-evaluated, however, when analyzing Table 4, it was observed the diversity of the study design. Each article worked with TiO_2_ NP unknown stability possibly holding large and sedimenting agglomerates, different TiO_2_ NPs exposure times, crystal structures and surface coatings, dissimilar measures for exposure doses (mass, area or particle number) that in practice result in a difficulty to convert into each other as well as did not adequately characterize particles' size and stability during cell exposure. From the 7 articles analyzed, six of them concluded that TiO_2_ NPs are non-irritating, non-corrosive and non-phototoxic (Park et al., 2011; Choi et al., 2014; Kato et al., 2014; Horie et al., 2016; Kim et al., 2016; Miyani and Hughes, 2016; Tang et al., 2018). What was noticed was that in the six articles analyzed, the toxicity of TiO_2_ NPs was evaluated using the MTT assay, which is reported in the literature to interfere with TiO_2_ NPs (Kroll and Hendrik, 2012; Ong et al., 2014). TiO_2_ NPs interference is attributed to the light adsorption properties of NP over the same spectral region used by MTT (Holder et al., 2012; Kroll and Hendrik, 2012; Ong et al., 2014). However, NPs also adsorb constituents of the assay on their surface that obstruct proper transformation of molecules, chemical reactions between the NPs and the test compounds can occur and even the release of metal ions from NPs can modify the mitochondrial catalytic activity of cells altering MTT reading (Kroll et al., 2009). Some authors reported that interference increases with increased NP concentration (Holder et al., 2012; Kroll and Hendrik, 2012; Ong et al., 2014), and emphasize that it is necessary to use concentrations of NPs that do not reduce MTT. It is important to stress that in the last years, extensive washing steps were introduced in the protocols and the possible interference of TiO_2_ NPs with the MTT assay was reduced and in some cases overcome (Kroll and Hendrik, 2012; Ong et al., 2014). However great care must be taken, because even with multiple washes or centrifugations, TiO_2_ NPs can remain adhered to the culture plate or even adsorbed on the surface of the cells. The contribution of NPs to the MTT light absorption signal depends also with the concentration of reduced MTT-formazan present in the MTTred/MTTox mixtures, suggesting different mechanisms of interference that cannot be predicted a priori (Kroll and Hendrik, 2012). It seems that redox- active metals with different sizes and coatings can change the magnitude of the reaction kinetics causing different levels of interference (Mello et al., 2020). Figure 4 shows an interference scheme of NP with the MTT assay. As it is possible to observe the properties of the NPs can generate artifacts and misinterpretation of the results (Holder et al., 2012; Kroll and Hendrik, 2012; Guadagnini et al., 2013; Lupu and Popescu, 2013; Ong et al., 2014; Lammel and Sturve, 2018). A recent article demonstrated that TiO_2_ NPs adsorbed on cell surface and those internalized by cells interfere with the fluorescence readout by reflecting/absorbing part of the incident and emitted light (Lammel and Sturve, 2018). This adds new complexity to NPs hazard evaluation since each specific cell type characteristics influence NPs internalization, intracellular trafficking and final destiny.

Besides MTT, other biological assays interfere with TiO_2_ NPs (see Table S9 in Supplementary Information). Lactate dehydrogenase (LDH) assay was also reported to interfere with TiO_2_ NPs, since they can adsorb or inactivate LDH protein (Holder et al., 2012; Kroll and Hendrik, 2012), as well as the Neutral Red (NR) (Guadagnini et al., 2013), Alamar Blue assay (Ong et al., 2014; Lammel and Sturve, 2018),5-Carboxyfluorescein diacetateacetoxymethylester (CFDA-AM) assay (Lammel and Sturve, 2018) and 2',7'-Dichlorofluorescein (DCF) (Kroll and Hendrik, 2012). Furthermore, interferences may be cumulative when for example two fluorometric assays are used on the same cells to quantify a unique endpoint.

Assessing the toxicity of TiO_2_ NPs, is not a trivial issue. Therefore, researchers need to use every time that it is possible validated protocols for skin irritation, corrosion and phototoxicity testing with 3D models. Apply concentration exposures that mimic real situations but at the same time concentrations below interfering levels. Every time that it is possible introduce centrifugation, several washes, or even removing supernatants, are recommended approaches to reduce interference. We believe that with effective washing procedures, we can expect that NPs cannot effectively and sufficiently interact with MTT in the way that could significantly alter the results, however we need to take in consideration that this approach introduces another concern regarding exposure characterization and applied dose metrics. Resuming assays adaptations have to be ascertained case by case with a series of control experiments for each NP to obtain reliable nanotoxicity data. We also suggest perform complementary tests such as the evaluation of cytokines, histopathology and evaluation of cell membrane integrity by detecting transepithelial electrical resistance (TEER), always working with specific controls.

A long time ago that the nanotoxicology community has been addressing technical questions, such as dosing issues, aggregation state of materials as a function of time. We believe to deliver a realistic risk assessment of NPs, it is necessary to identify the key physicochemical characteristics that can foresee toxicological results, work with exposure conditions that mimic a real situation and characterize NPs and their interactions with biological systems (example: protein corona, its transformational capacity in the biological system). Very subtle alterations in the properties of NPs can completely alter protein corona that gets absorbed onto the NPs resulting in surprising changes *in vivo*. As the degree of interference may be relevant depending on the optical properties of the NPs, stability of NP, cytotoxicity testing and the type of fluorometric endpoint assay, it is encouraged to use more than one *in vitro* assay (for example flow cytometry is considered the method with less interference with NPs), specifically with different detection methods and use adequate controls (as negative controls, it would be advisable to separately test the dispersant agents used as NPs stabilizers under the same conditions). Adequate reference materials are needed in toxicological studies and efforts should be done in order to evaluate more than cell viability (ex: cell cycle) (Singh et al., 2019).

## Conclusion

Through the data obtained by this systematic review, TiO_2_ NPs was considered non-irritant, non-corrosive and non-phototoxic for the 3D skin model, regardless of the crystalline type, and the size of the NPs studied. We can conclude that skin 3D models can be used to test the hazard effect of TiO_2_ NPs, however, we emphasize the need of standardized protocols with efficient washing procedures to remove the NP from the surface of 3D skin model to avoid potential interfere of TiO_2_ NPs with the assay.

## Data Availability Statement

All datasets generated for this study are included in the article/Supplementary Material.

## Author Contributions

PS, LG, RC, AR, and JG contributed to study concept and design. PS, LG, and RC contributed to the literature search and data collection. PS, LG, RC, DS, ML, AR, and JG contributed to data analysis. PS, RC, and AR wrote the paper. All authors contributed to a critical revision of the manuscript.

## Conflict of Interest

The authors declare that the research was conducted in the absence of any commercial or financial relationships that could be construed as a potential conflict of interest.
