# Supplementary material for: Toxicity Evaluation of TiO2 Nanoparticles on the 3D Skin Model: A Systematic Review
Source: Front Bioeng Biotechnol. 2020 Jun 10;8:575. doi: 10.3389/fbioe.2020.00575 (PMC7298140; doi:10.3389/fbioe.2020.00575)
Supplement: Supplementary file 1 [file Data_Sheet_1.docx]

**Supplementary Information**

**Toxicity evaluation of TiO_2_**

**nanoparticles on 3D skin model:**

**A Systematic Review**

Priscila Laviola Sanches^1,3^, Luths Raquel de Oliveira Geaquinto^2,3^, Rebecca Cruz^4^, Desirée Cigaran Schuck^5^, Márcio Lorencini^5^, José Mauro Granjeiro^1,2,3,4*^ and Ana Rosa Lopes Ribeiro^1,2,3,6*†^

^1^Postgraduate Program in Translational Biomedicine, University of Grande Rio, Duque de Caxias, Brazil, ^2^Postgraduate Program in Biotechnology, National Institute of Metrology Quality and Technology, Duque de Caxias, Brazil, ^3^Metrology Directorate Applied to Life Sciences, National Institute of Metrology, Quality and Technology, Duque de Caxias, Brazil, ^4^Federal Fluminense University, Niterói, Brazil, ^5^Pesquisa e Desenvolvimento, Grupo Boticário, Curitiba, Brazil, ^6^3Bs Research Group, Biomaterials, Biodegradables and Biomimetics, Dept. of Polymer Engineering, University of Minho, Braga, Portugal.

Corresponding authors: Ana Ribeiro and José Mauro Granjeiro

Correspondence: [analopes0781@gmail.com](mailto:analopes0781@gmail.com) or [jmgranjeiro@gmail.com](mailto:jmgranjeiro@gmail.com)

**Table S1:** Simulation of a work with 100% of the criteria matched


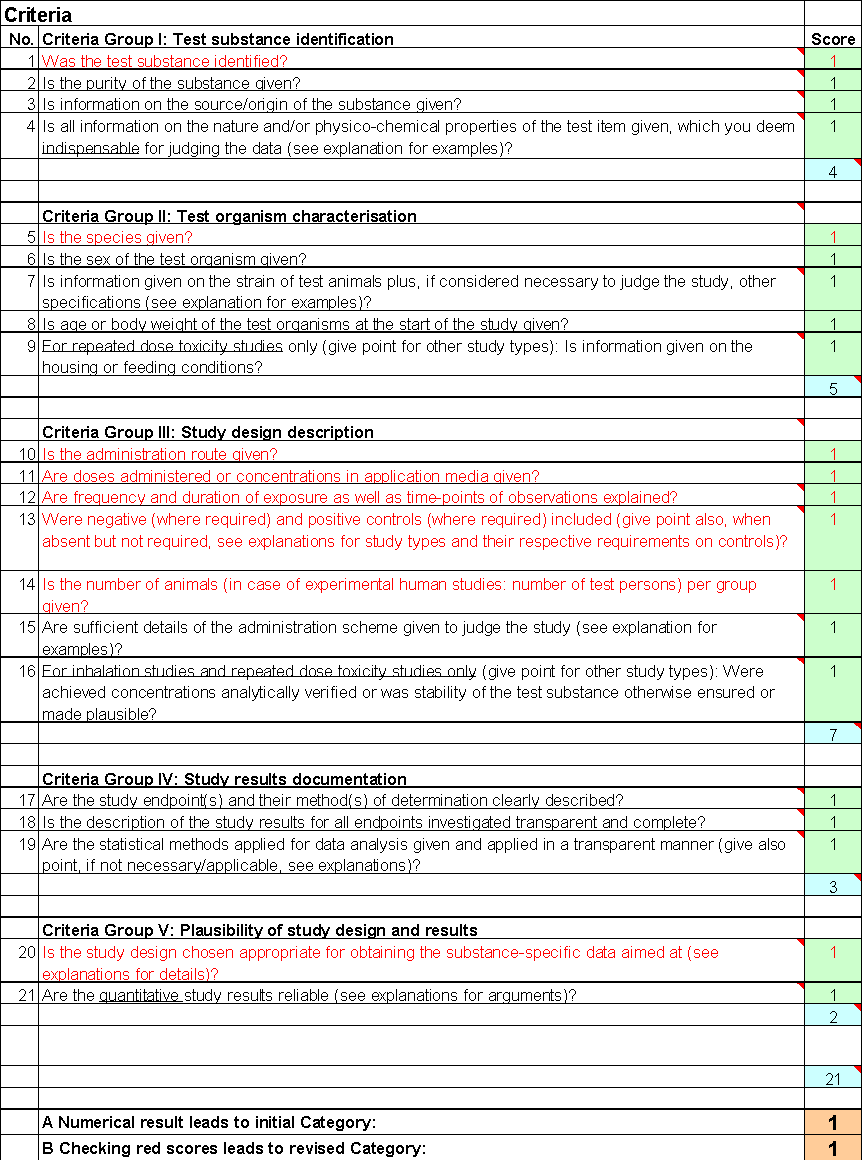


**Table S2:** Criteria evaluated using ToxTool. Evaluation of the article by Park et al. (2011)
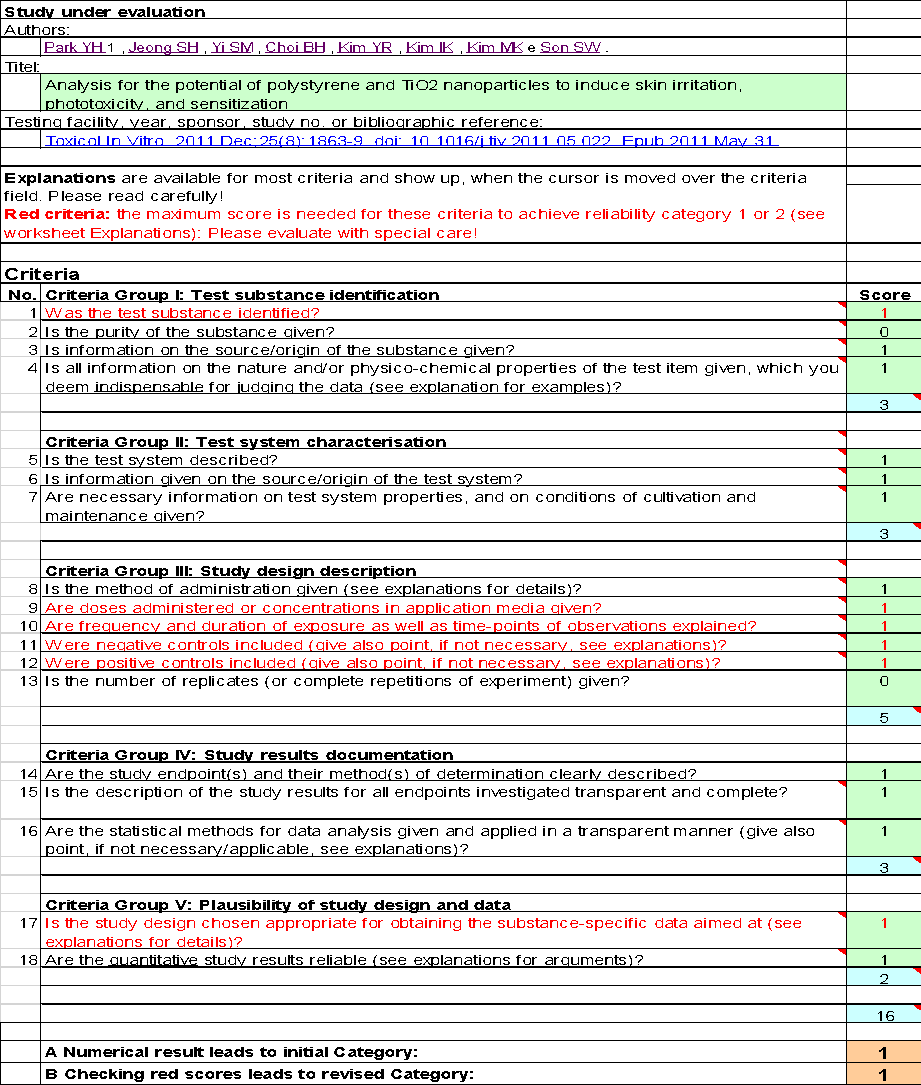


**Table S3:** Criteria evaluated using ToxTool. Evaluation of the article by Choi et al. (2014)


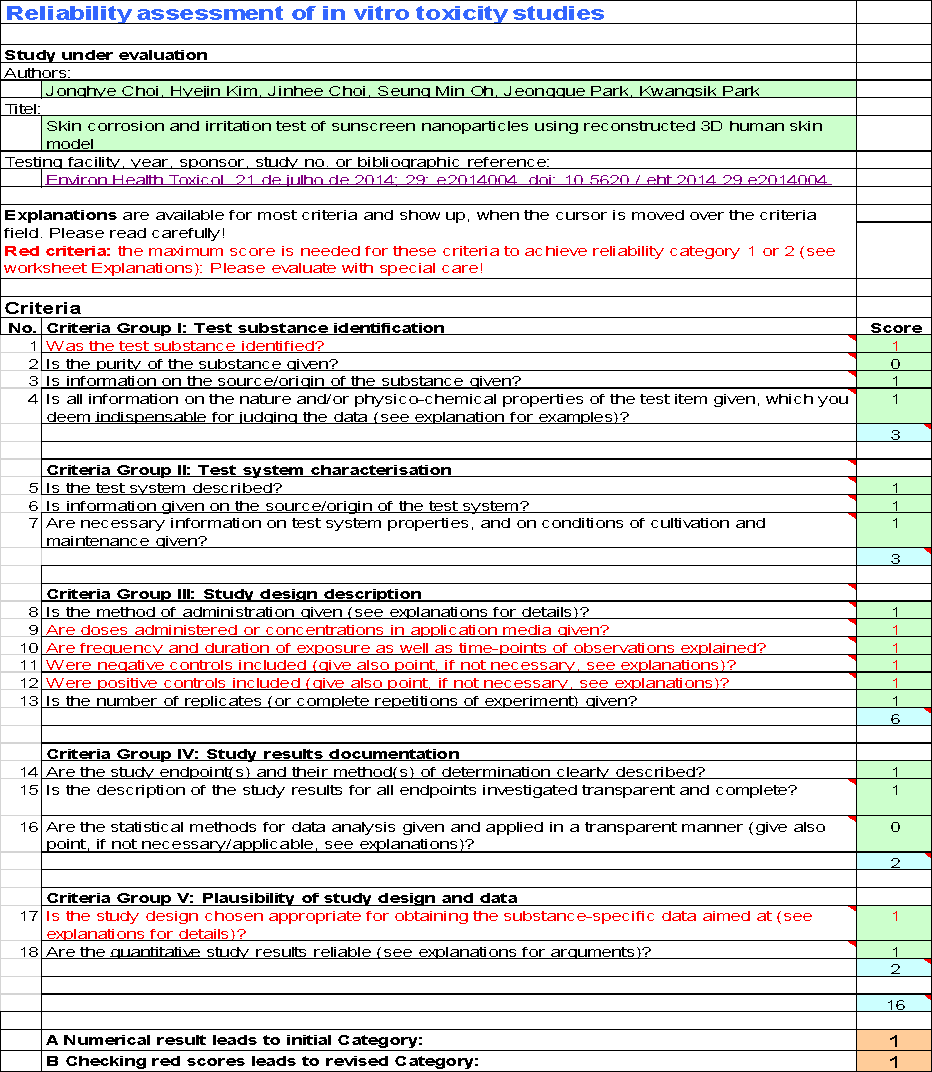


**Table S4:** Criteria evaluated using ToxTool. Evaluation of the article by Kato et al. (2014)


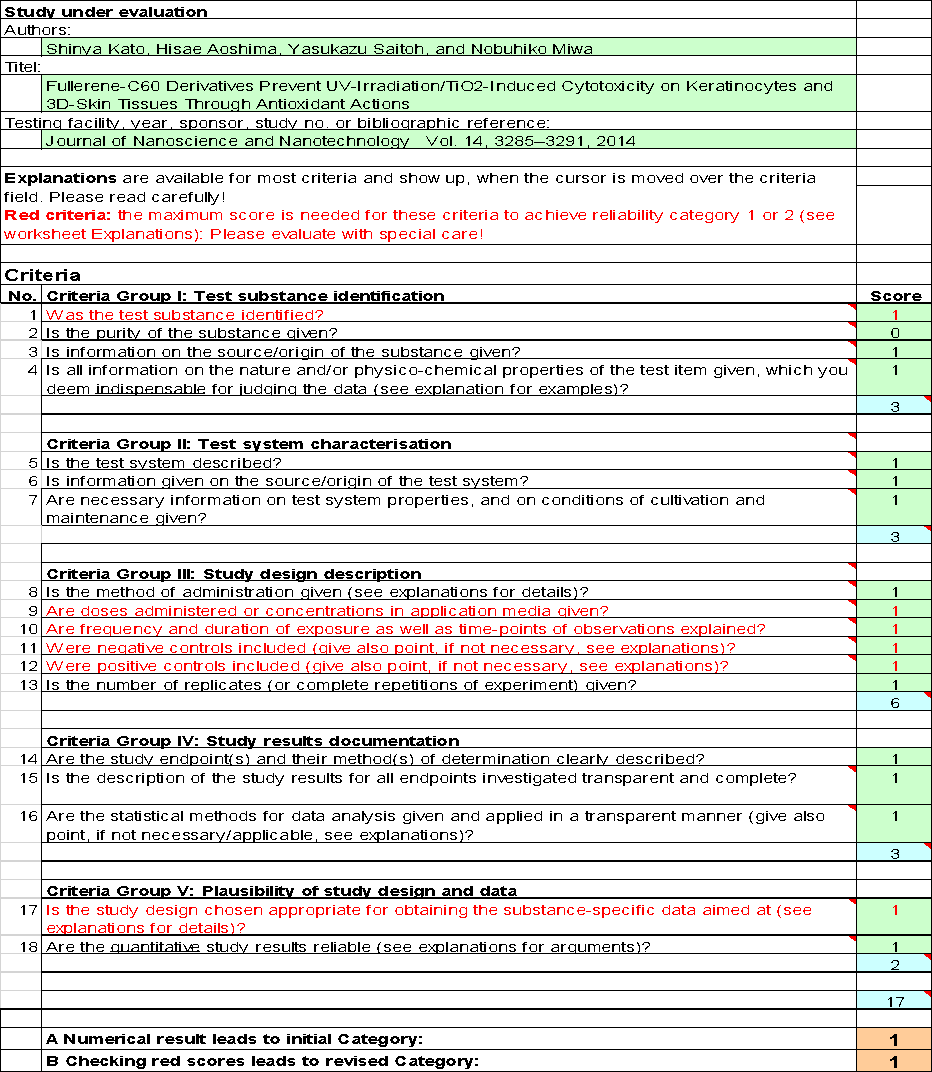


**Table S5:** Criteria evaluated using ToxTool. Evaluation of the article by Horie et al. (2016)


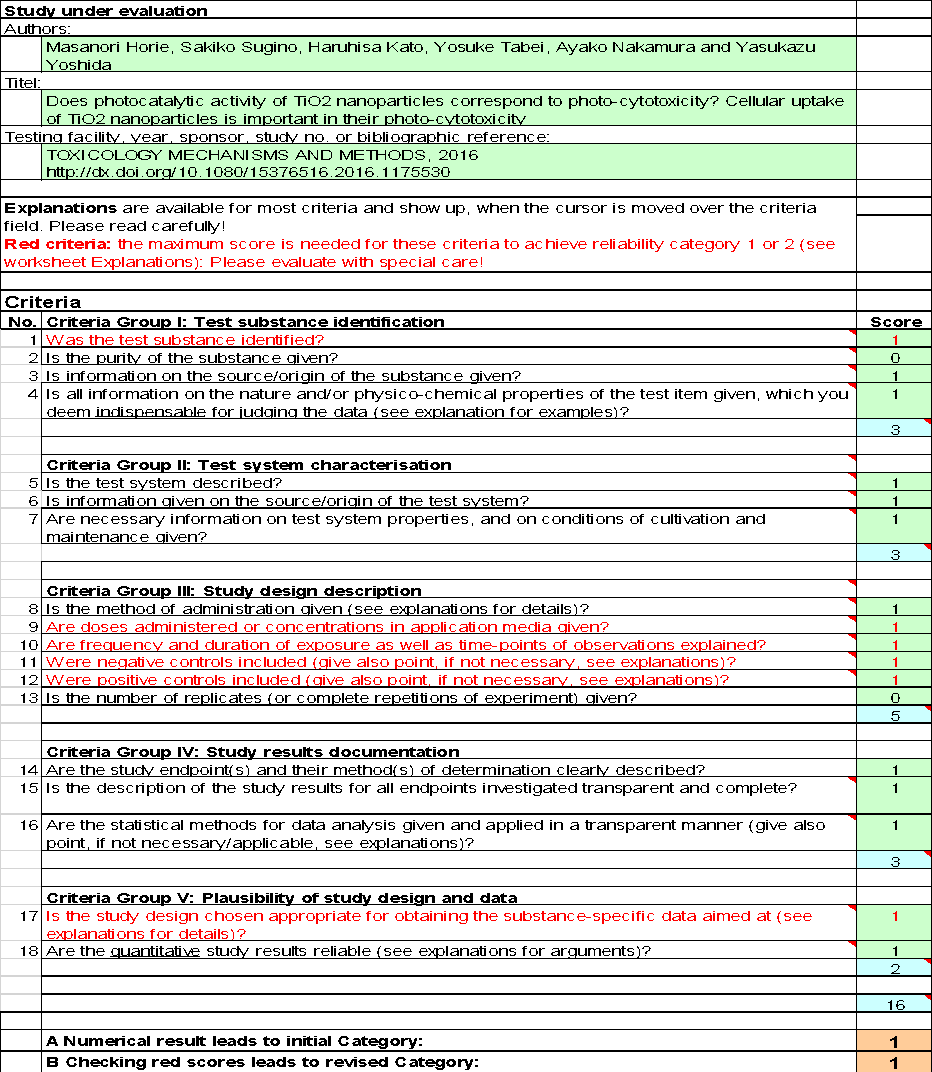


**Table S6:** Criteria evaluated using ToxTool. Evaluation of the article by Miyani and Hughes (2016)


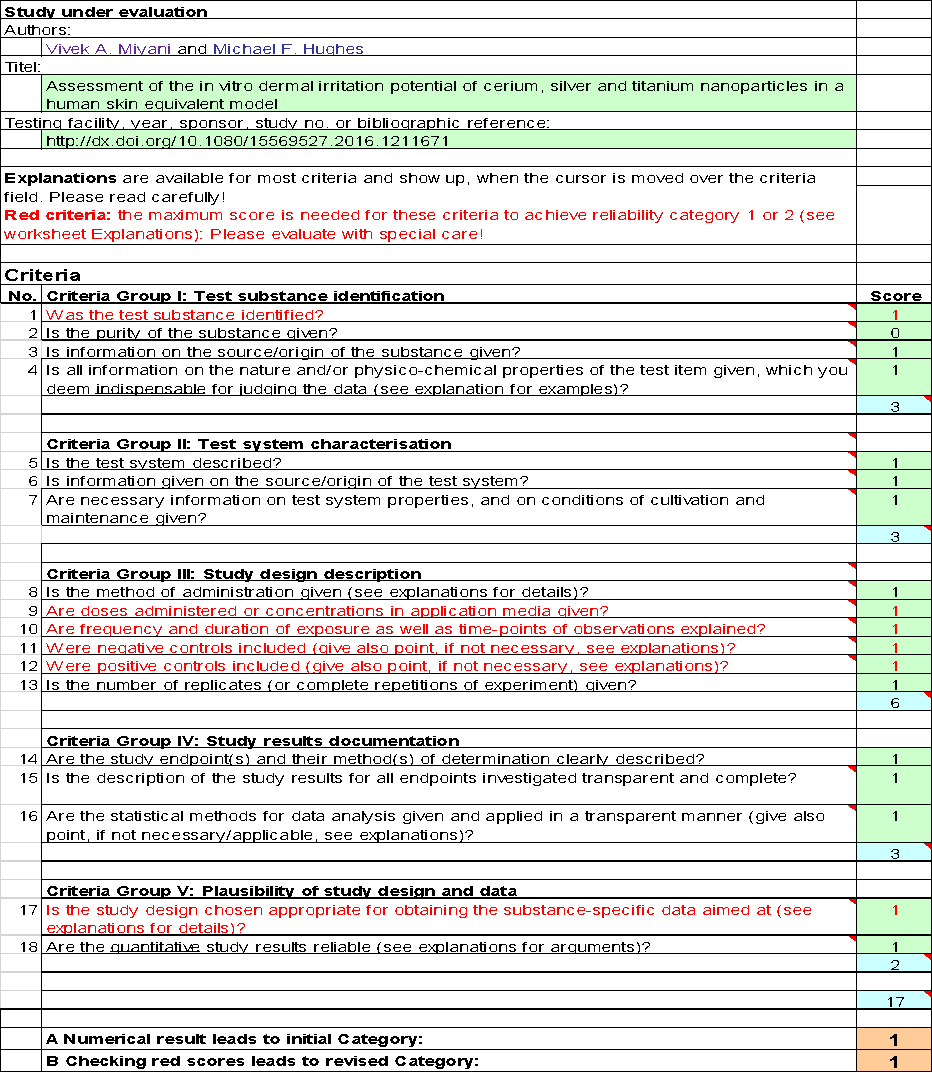


**Table S7:** Criteria evaluated using ToxTool. Evaluation of the article by Kim et al*.* (2016)
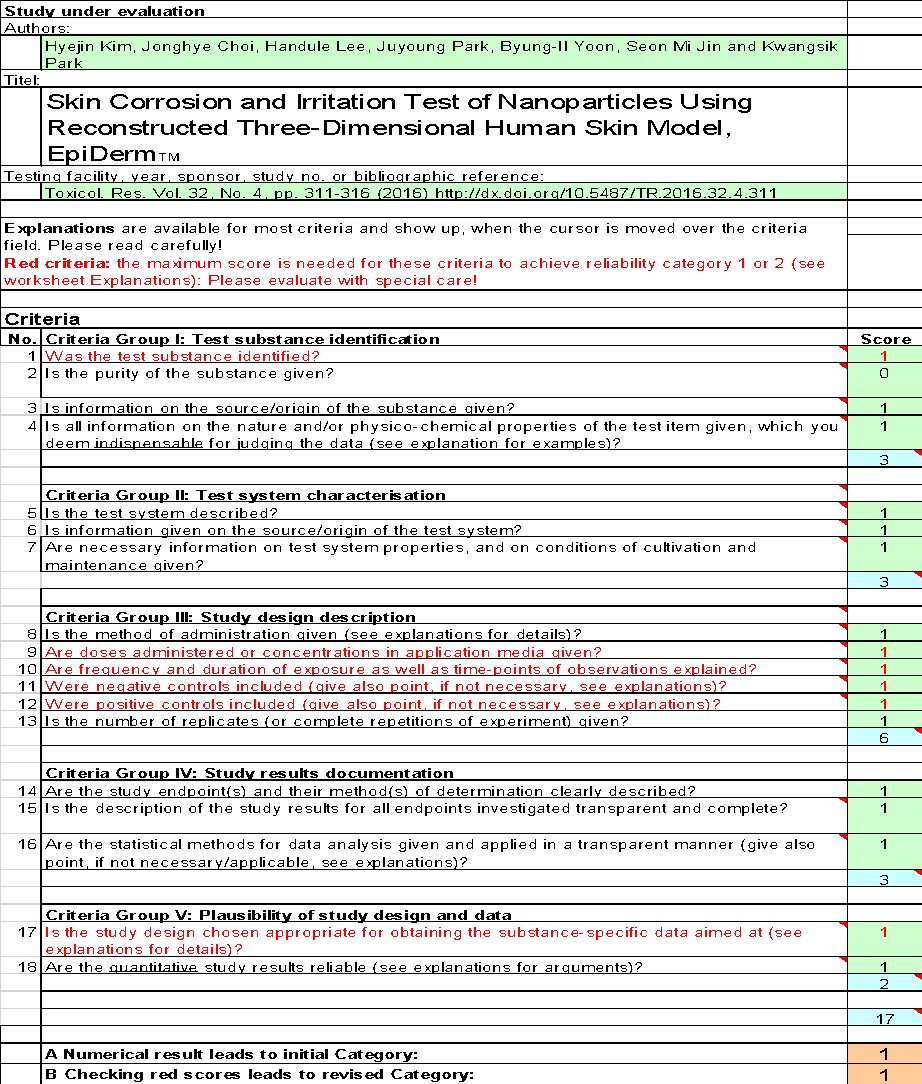


**Table S8:** Criteria evaluated using ToxTool. Evaluation of the article by Tang et al. (2018)
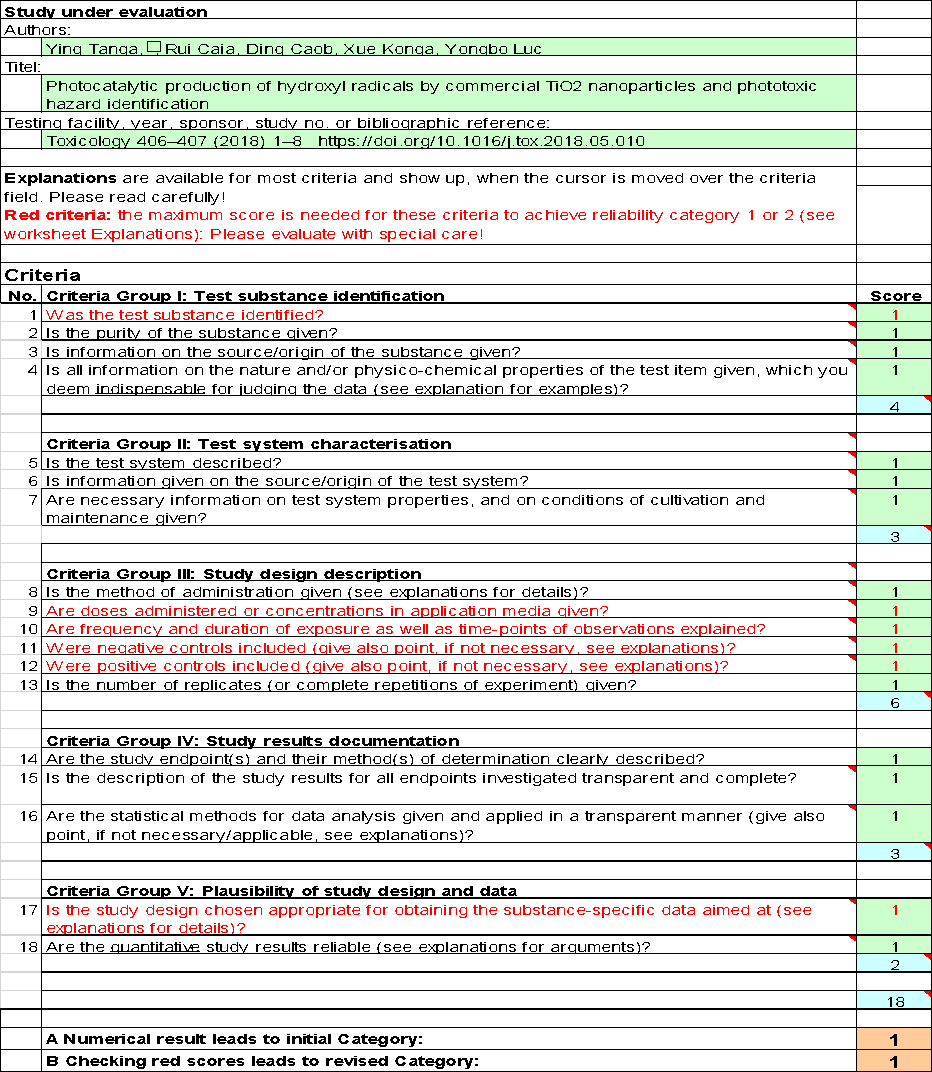


**Table S9:** Assays described in the literature for interfering with TiO_2_ NPs

| **AUTHOR** | **ASSAY** |
| --- | --- |
| Kroll et al. (2012) | DCF; MTT; LDH |
| Ong et al. (2014) | Alamar Blue assay |
| Holder et al. (2012) | MTT; LDH |
| Lammel and Sturve (2018) | Alamar Blue assay; CFDA-AM assay |
| Lupu and Popescu (2013) | MTT |
| Guadagnini et al. (2013) | NR; MTT |
