# Supplementary figures and images for: Toxicity Evaluation of TiO2 Nanoparticles on the 3D Skin Model: A Systematic Review
Source: Front Bioeng Biotechnol. 2020 Jun 10;8:575. doi: 10.3389/fbioe.2020.00575 (PMC7298140; doi:10.3389/fbioe.2020.00575)

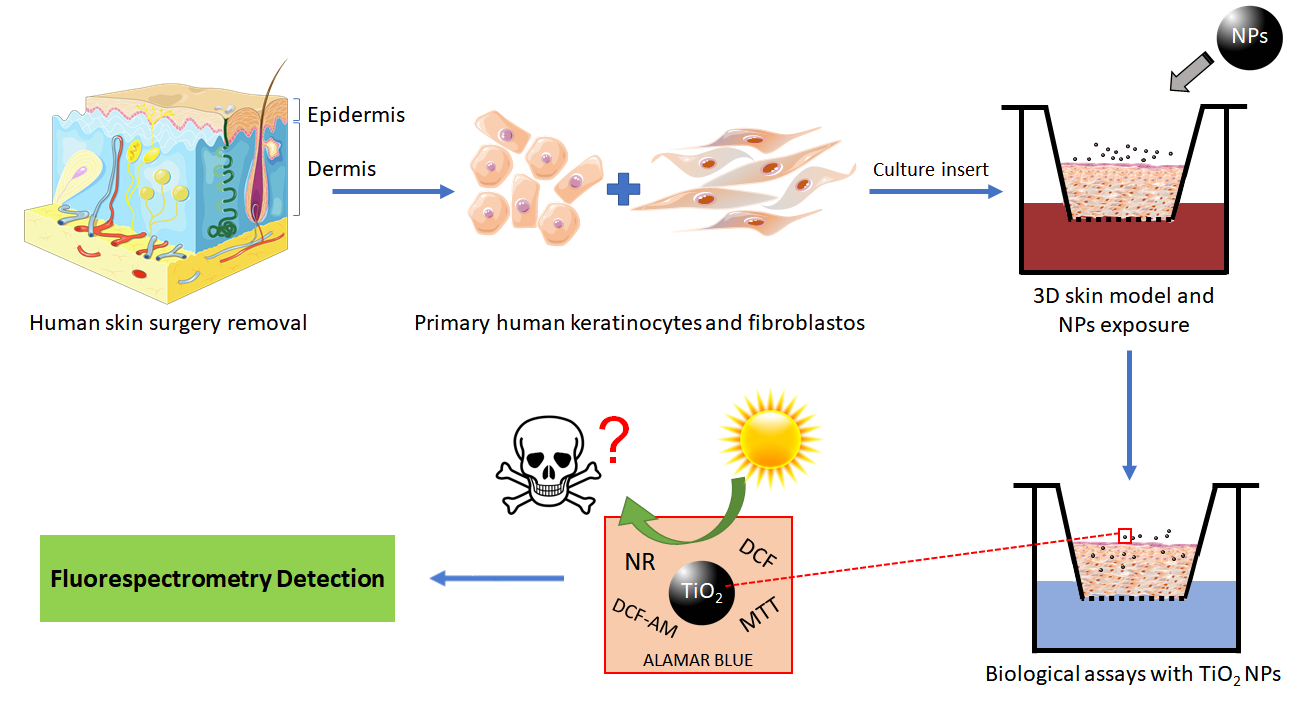

Supplement: Supplementary file 2 [file Image_1.PNG]
